# Local and genetic determinants of vascular endothelial growth factor expression in advanced proliferative diabetic retinopathy

**Published:** 2008-07-30

**Authors:** Mojca Globočnik Petrovič, Peter Korošec, Mitja Košnik, Joško Osredkar, Marko Hawlina, Borut Peterlin, Daniel Petrovič

**Affiliations:** 1 Eye Clinic, University Medical Centre Ljubljana, Ljubljana, Slovenia; 2 University Clinic of Respiratory and Allergic Diseases, Golnik, Slovenia; 3 University Institute for Clinical Chemistry and Biochemistry, University Medical Centre Ljubljana, Ljubljana, Slovenia; 4 Division of Medical Genetics, Department of Obstetrics and Gynecology, University Medical Centre Ljubljana, Ljubljana, Slovenia; 5 Institute of Histology and Embryology, Medical Faculty, University of Ljubljana, Ljubljana, Slovenia

## Abstract

**Purpose:**

In proliferative diabetic retinopathy (PDR) and other angiogenesis-associated diseases, increased levels of cytokines, inflammatory cells, growth factors, and angiogenic factors are present. Vascular endothelial growth factor (VEGF) appears to play a central role in mediating microvascular pathology in PDR. The purpose of the present study was to search for the association between the –634 C/G polymorphism of the *VEGF* gene and PDR. Moreover, it was hoped to determine whether serum and vitreous levels of VEGF are affected by genetic factors.

**Methods:**

This cross-sectional case-control study enrolled 349 unrelated Slovene subjects (Caucasians) with type 2 diabetes mellitus. The case group consisted of 206 patients with an advanced form of PDR and for whom vitrectomy was performed, and the control group had 143 patients who had no clinical signs of diabetic retinopathy but did have type 2 diabetes of more than 10 years duration. To analyze the genotype distribution we had to compare the genotype frequencies in diabetics with PDR (cases, n=206) and diabetics without diabetic retinopathy (control group, n=143). Additionally, to evaluate the effect of diabetes on the VEGF serum levels 2 groups, diabetics and non diabetics, were compared. First group were diabetics (diabetics with PDR, n=104), and second group were 29 subjects without diabetes.

**Results:**

The –634 C/G *VEGF* polymorphism was not associated with PDR. Mean serum and vitreous levels of VEGF were statistically significantly higher in PDR in comparison to the control group. Moreover, significantly higher serum and vitreous levels of VEGF were demonstrated in diabetics with the CC genotype compared to those with the other (CG + GG) genotypes.

**Conclusions:**

VEGF is an important cytokine in PDR. Despite the effect of the –634 C/G *VEGF* polymorphism on serum and vitreous levels of VEGF in PDR, it failed to contribute to the genetic susceptibility to PDR.

## Introduction

In proliferative diabetic retinopathy (PDR) and other angiogenesis-associated diseases, increased levels of cytokines, inflammatory cells, growth factors, and angiogenic factors are present [1-5]. Vascular endothelial growth factor (VEGF) appears to play a central role in mediating microvascular pathology in PDR. VEGF is capable of inducing the earliest changes in diabetic retinopathy such as leukostasis and blood-retinal barrier breakdown [6,7] as well as macular edema and neovascularization in progression of diabetic retinopathy [1]. In the vitreous of patients with PDR, VEGF levels have been found to be increased [1,2,4,5]. Although diabetes duration and inadequate glycemic control are important risk factors in the development of PDR, genetic factors may play a significant role in the pathogenesis of PDR [8,9]. There is considerable variation in VEGF expression among individuals, with several different polymorphisms being reported [10]. The *VEGF* 634 C/G (rs2010963) polymorphism in the 5′-untranslated region has been reported to be associated with variations in VEGF serum concentrations and with a susceptibility to disorders, such as diabetic retinopathy, diabetic nephropathy, and cardiovascular diseases [8,10-13].

To investigate the impact of genetic polymorphisms of *VEGF* on PDR in a Slovenian population (Caucasians) with type 2 diabetes, we searched for the association between the -634 C/G *VEGF* polymorphism and PDR in subjects with type 2 diabetes. Moreover, the aim of the study was to determine the serum and vitreous levels of VEGF of patients with PDR, and whether serum and vitreous levels of VEGF are affected by genetic factors.

## Methods

### Patients

This cross-sectional case-control study enrolled 349 (age range 35 to 87 years; 152 men, 197 women) unrelated Slovene subjects (Caucasians) with type 2 diabetes mellitus who had a defined ophthalmologic status. Patients were classified as having type 2 diabetes according to the current American Diabetes Association criteria for the diagnosis and classification of diabetes [14]. Patients were recruited from the Eye Clinic of the University Medical Centre Ljubljana between January 2002 and April 2007. Fundus examination was performed by a senior ophthalmologist (M.G.P.) after pupil dilatation (tropicamide and phenylephrine 2.5%) using slit-lamp biomicroscopy with non-contact lens, and was electronically documented with a 50°-angle fundus camera (Topcon-TRC 40-IX; Topcon, Tokyo, Japan). Staging of diabetic retinopathy was determined according to the Early Treatment Diabetic Retinopathy Study Research Group retinopathy severity scale [15].

The study group consisted of 206 patients with an advanced form of PDR (new vessel formation as well as fibrous proliferation with or without vitreous hemorrhage) in whom vitrectomy was indicated and performed due to vitreous hemorrhage, macular detachment, or macular threatening detachment. The control group consisted of 143 patients who had type 2 diabetes of more than 10 years duration but had no clinical signs of diabetic retinopathy.

In 68 out of 206 patients with PDR (71 eyes) 0.3 ml vitreous fluid samples were obtained by vitreoretinal surgery. The study excluded patients who had previous vitrectomy, neovascularization of no diabetic etiology, recent vitreous hemorrhage (less than two months), or a history of ocular inflammation and photocoagulation in the preceding three months.

Macular edema was defined as being clinically significant based on observations rendered by the Early Treatment Diabetic Retinopathy Study [16]. PDR was found to be active in 54 eyes and inactive in 17 eyes, according to the method reported by Aiello and associates [1]. Neovascularization was considered to be active if new vessels were perfused, multibranching iridic, or preretinal capillaries; it was considered to be inactive if previously documented active proliferation had regressed fully or if only nonperfused gliotic vessels or fibrosis were present [1]. The extent of retinal laser photocoagulation was classified into three grades: grade 1) no photocoagulation; grade 2) focal photocoagulation; and grade 3) panretinal photocoagulation (defined as extensive photocoagulation in all four quadrants of the retina).

### Sample collection

Fasting serum VEGF levels were analyzed in 104 out 206 of patients with PDR and in 29 patients without diabetes. Vitreous fluid samples (0.3 ml) were obtained by vitreoretinal surgery (from the midvitreous at the initial stage of vitrectomy by aspiration into a 2 ml syringe attached to the vitreous counter before starting intravitreal infusion of balanced salt solution) from 71 eyes of 68 consecutive patients with PDR, and from 17 eyes of 17 consecutive nondiabetic patients in whom vitrectomy was performed because of idiopatic macular hole [17]. Vitreous was cut and aspirated into a 2 ml syringe attached to the vitreous cutter before starting intravitreal infusion of balanced salt solution.

### Single nucleotide polymorphism –634 C/G gene polymorphisms and diabetic retinopathy

The –634 C/G VEGF polymorphism was evaluated as described previously [8]. Genotype determination: Genomic DNA was isolated from peripheral blood lymphocytes by standard methods and stored at -20 °C. Genotyping was carried out by polymerase chain reaction-restriction fragment length polymorphism (PCR-RFLP) analysis. In brief, each PCR reaction (20 μl) contained 0.4 μl 10 mM dNTP (Gibco), 2 μl 10-X PCR buffer (Gibco), 1.2 μl 25 mM MgCl2 (final concentration 1,5 mM), 1 μl 10 μM of each primer (5′-TTG CTT GCC ATT CCC CAC TTG A-3′, 5′-CCG AAG CGA GAA CAG CCC AGA A-3′), 0.7 μl (500 ng) DNA, 12.5 μl H_2_0, 1 μl 5% DMSO, 0,5 unit of AmliTaq Gold DNA-polymerase (Applied Biosystems). Cycling parameters were as follows: 3 min 94 °C for primary denaturation, followed by 30 cycles of 1 minute 94 °C and 1 minute 59 °C, 1 minute 72 °C. After digestion for 16 hours at 65 °C by 1 unit of the restriction enzyme BsmFI, the restriction products were electrophoresed on a 2.0% agarose gel and then visualized using ethidium bromide staining. The -634G allele result in the gain of a BsmFI site. Two investigators (B.P., D.P.), blinded for case or control status of the DNA sample, performed the genotype classification.

### VEGF serum concentration assay

For the determination of fasting serum VEGF concentration (isoform VEGF 165), we used a solid phase sandwich ELISA, which involved two kinds of highly specific antibodies (hVEGF Assay Kit; IBL Co., Ltd., Takasaki-shi, Gunma, Japan). In our study the respective coefficient of variation (CV; %) were between 3 and 5.5 for interassay measurements, and between 2.6 and 5.3 for intraassay measurements.

The vitreous VEGF concentration was determined with the cytometric bead array method (CBA; BD Biosciences, San Diego, CA). The samples were collected in sterile tubes, rapidly frozen and then stored at –80 C until analyzed. VEGF concentrations were measured using CBA (BD Biosciences). The VEGF capture beads were mixed with phycoerythrin – conjugated detection antibodies. These capture beads were incubated with recombinant standards or test samples (vitreous) to form sandwich complexes. Two-color flow cytometric analysis was performed using a FACSCalibur flow cytometer (BD Biosciences). Data were acquired and analyzed using Becton Dickinson Cytometric Bead Array CBA software, and concentrations were determined from the standard curves, plotting recombinant calibrator concentration versus FL-2 mean fluorescence intensity.The use of this method made it possible to detect two of the four VEGF isoforms (VEGF 121 and VEGF 165).

### Statistical analysis

We used a nonparametric Mann–Whitney test and Kruskal–Wallis test for multiple group comparison or the Student *t*-test as appropriate. The χ^2^ test was used to compare discrete variables and to compare genotype distributions. In addition, all variables that showed significant differences by univariate methods (χ^2^ test, unpaired Student *t* test) were analyzed together in a logistic regression analysis. Statistical analysis was performed using the SPSS program for Windows version 14 (SPSS Inc. Chicago, IL).

## Results

The characteristics of the cases and control subjects are listed in Table 1. Cases had earlier onset of diabetes (45.2±12.1 years vs. 53.3±12.1 years; p<0.001) and longer duration (19.2±8.7 years vs. 16.5±6.6; p=0.009) of type 2 diabetes compared to controls diabetics without diabetic retinopathy). Additionally, they had higher incidence of insulin therapy than the controls (diabetics without diabetic retinopathy). There were no significant differences in hypertension, smoking, total, high density lipoprotein (HDL) cholesterol, and low density lipoprotein (LDL) cholesterol, and triglyceride levels between the cases and controls.

**Table 1:** Characteristics of patients with proliferative diabetic retinopathy (PDR; cases) and patients without diabetic retinopathy (controls).

**Characteristics**	**PDR n (%)**	**Controls n (%)**	**p value**
Number	206	143	
Age (years)	65.0 ± 9.9	66.9 ± 11.5	0.2
Male sex (%)	95 (46.1)	57 (39.9)	0.2
Duration of diabetes (years)	19.2 ± 8.7	16.5 ± 6.6	0.009
Patients on insulin therapy (%)	154 (74.8)	66 (46.1)	<0.001
Age of diabetes onset	45.2 ± 12.1	53.3 ± 12.1	<0.001
HbA_1c_ (%)	8.1 ± 1.6	8.2 ± 1.6	0.3
Systolic blood pressure (mmHg)	144 ± 24	145 ± 20	0.7
Diastolic blood pressure (mmHg)	85 ± 12	84 ± 9	0.4
Body mass index (kg/m^2^)	28.1 ± 4.4	27.7 ± 4.4	0.9
History of hypertension (%)	159 (77.2)	100 (70)	0.1
Smokers (%)	25 (12.1)	15 (10.5)	0.9
Total cholesterol (mmol/l)	5.4 ± 1.2	5.5 ± 1.2	0.1
HDL cholesterol (mmol/l)	1.1 ± 0.4	1.2 ± 0.4	0.1
LDL cholesterol (mmol/l)	3.1 ± 1.0	3.2 ± 0.9	0.2
Triglycerides (mmol/l)	2.2 ± 1.3	2.6 ± 1.9	0.1

Cases had earlier onset of diabetes and longer duration of type 2 diabetes compared to controls (diabetics without diabetic retinopathy). Additionally, they had higher incidence of insulin therapy than the controls (diabetics without diabetic retinopathy). Numbers are given as n (%). Abbreviations: HDL, high density lipoprotein, LDL, low density lipoprotein.

### Single nucleotide polymorphism –634 C/G *VEGF* polymorphisms and PDF

The VEGF genotype distributions in cases and controls (Table 2) were compatible with Hardy–Weinberg expectations (–634 C/G: cases χ^2^=1.21, p=0.27; –634 C/G: controls χ^2^=0.29, p=0.59). The –634 C/G *VEGF* polymorphism was not associated with PDR in a group of Caucasian subjects with type 2 diabetes (Table 2).

**Table 2:** Distribution of vascular endothelial growth factor genotypes/alleles in patients with proliferative diabetic retinopathy (cases) and in those without diabetic retinopathy (controls).

**Genotype/allele** **(-634 C/G polymorphism)**	**Cases** **n (%)**	**Controls** **n (%)**	**p**	**Odds ratio** **(95% CI)**
Genotype CC	24 (11.6)	15 (10.5)	0. 7^1^	1.1 (0.5–2.1)^1^
Genotype CG	103 (50.0)	67 (46.9)		
Genotype GG	79 (38.4) 206	61 (42.6) 143		
C allele	151 (36.7)	97 (33.9)	0. 5^2^	
G allele	261 (63.3)	189 (66.1)		

Distribution of the –634 C/G vascular endothelial growth factor genotypes/alleles was compared between cases and controls. The frequencies of genotypes and alleles are shown in percentage and as seen in the Table 3 there are no significant differences between cases and controls in either the frequency of the CC genotype (CC versus CG plus GG: odds ratio 1.1, 95% confidence interval 0.5–2.1; p value 0.7). ^1^p-value and odds ratio (CC versus CG plus GG), ^2^p value for allele frequency.

The variables that showed significant differences by χ^2^ test and unpaired Student *t* test (insulin therapy, diabetes duration, age of diabetes onset) plus the *VEGF* polymorphism were analyzed together in a logistic regression analysis. In the logistic regression model (diabetes duration, age of diabetes onset, incidence of insulin therapy, and the *VEGF* polymorphism) the CC genotype of the *VEGF* polymorphism was not an independent risk factor for PDR (Table 3).

**Table 3:** Logistic regression analysis for the association with proliferative diabetic retinopathy among type 2 diabetic patients.

**Risk factor**	Odds ratio (95% CI)^*^	**p**
CC genotype^**^	1.1 (0.7–1.6)	0.7
Patients on insulin therapy (%)	3.3 (1.9–5.7)	< 0.001
Age of diabetes onset	1.06 (1.029–1.09)	< 0.001
Duration of diabetes (years)	1.025 (0.983–1.068)	0.2

The asterisk indicates CI is confidence interval; the double asterisk shows the *VEGF* -634 C/G polymorphism. In the logistic regression model diabetes duration, age of diabetes onset, and incidence of insulin therapy were demonstrated to be an independent risk factor for PDR, whereas the CC genotype of the VEGF polymorphism was not an independent risk factor for PDR. The odd ratio for the CC genotype of the –634 C/G VEGF gene polymorphism is 1.1, 95% confidence interval is 0.7-1.6; p=0.7.

### VEGF serum levels

Fasting serum VEGF levels in 104 PDR patients (55.3±25.1 ng/l) were significantly higher from those of 29 controls without diabetes (13.8±10.2 ng/l; p<0.001). To analyze the genotype distribution we had to compare the genotype frequencies in diabetics with PDR (cases, n=206) and diabetics without diabetic retinopathy (control group, n=143). However, to evaluate the effect of diabetes on the VEGF serum levels 2 groups, diabetics and non-diabetics, were compared. First group were diabetics (diabetics with PDR, n=104), and second group were 29 subjects without diabetes.

Moreover, we found significantly higher VEGF serum levels in 24 PDR patients with the CC genotype (60.4±32.1 ng/l) compared to 80 PDR patients with the other (CG + GG) genotypes (44.1±23.5 ng/l; p<0.01); 24 PDR patients with the CC genotype did not differ in clinical parameters (age, diabetes duration, age of diabetes onset, lipids, blood pressure, incidence of insulin therapy, sex distribution, incidence of smoking) from 80 PDR patients with the other genotypes (data not shown).

We failed, however, to demonstrate statistically significant differences in fasting serum VEGF levels between patients with macular edema (62.1±39.1 ng/l ) and patients without macular edema (54.4±34.7 ng/l; p=0.4). Moreover, we failed to demonstrate statistically significant differences in fasting serum VEGF levels between patients with active neovascularization (56.3±41.8 ng/l ) and patients without active neovascularization (62.8±26.6 ng/l; p=0.5)

### VEGF vitreous levels

Vitreous VEGF levels were analyzed in 71 eyes of 68 patients with PDR, and from 17 eyes of 17 patients without diabetes in whom vitrectomy was performed due to idiopatic macular hole [17]. Mean vitreous levels of VEGF were statistically significantly higher in diabetic patients with PDR (5283.51±5274.12 pg/ml )in comparison to the control group (17.40±12.57 pg/ml; p<0.001).

Vitreous VEGF levels were significantly higher in 12 eyes from 12 patients with the CC genotype (6061.52±5341.69 pg/ml ) compared to 59 eyes from 56 subjects with the other (CG + GG) genotypes (3026.43±2842.17 pg/ml; p<0.01); 12 PDR eyes from 12 PDR patients with the CC genotype did not differ in clinical parameters (age, diabetes duration, age of diabetes onset, lipids, blood pressure, incidence of insulin therapy, sex distribution, incidence of smoking, and incidence of panretinal photocoagulation) from 59 PDR eyes from 56 patients with the other genotypes (data not shown).

Macular edema was present in 37 out of 71 eyes with PDR. Significantly higher vitreous VEGF levels were demonstrated in the eyes with macular edema (6567.96±5780.66 pg/ml) compared to 34 eyes without macular edema (3885.73±4322.94 pg/ml; p=0.02).

Additionally, we wanted to compare vitreous VEGF levels between active stage of PDR (54 eyes out of 71 in whom vitrectomy was performed) and inactive stage of PDR (17 eyes out of 71 in whom vitrectomy was performed). Statistically significant differences between active (n=54) and inactive stages (n=17) of PDR were demonstrated in vitreous levels of VEGF (6666.25±5341.69 pg/ml versus 891.28±478.24 pg/ml; p<0.001).

The extent of laser photocoagulation was related to the vitreous level of VEGF. In the group of 13 eyes with no laser photocoagulation (7541.46±6323.89 pg/ml), the VEGF level was significantly higher compared to the group of 32 eyes with incomplete photocoagulation (6631.64±5543.10 pg/ml) or the group of 26 eyes with photocoagulation in all four quadrants (2495.3±2743.56 pg/ml; p=0.001).

## Discussion

In our study we analyzed the *VEGF* –634 C/G polymorphism as a potential genetic marker of PDR. The *VEGF* –634 C/G polymorphism was not associated with PDR in a group of Caucasians with type 2 diabetes. In accordance with our study, Awata and coworkers [8] failed to demonstrate an association between the CC genotype of the *VEGF* –634 C/G polymorphism and PDR, but they reported an association with diabetic retinopathy. They compared 70 Japanese patients with PDR (22.9% ) to 118 Japanese diabetics without diabetic retinopathy (10.3%), and they failed to demonstrate a statistically significant difference (p=0.081) in the frequency of the CC genotype [8]. Suganthalakshmi and coworkers [12] compared 120 Indian patients with diabetic retinopathy (17.5%) to 90 diabetics without diabetic retinopathy (30%), and they failed to demonstrate a statistically significant difference in the frequency of the CC genotype (p=n.s.). Moreover, Awata and coworkers [13] have recently demonstrated that the *VEGF C-634G* polymorphism is a genetic risk factor for diabetic macular edema as well as diabetic retinopathy.

Serum VEGF levels were found to be affected by genetic factors (the *VEGF* –634 C/G polymorphism). We analyzed fasting serum VEGF levels in 104 PDR patients and found significantly higher VEGF serum levels in 24 patietns with the CC genotype compared to 80 patients with the other (CG + GG) genotypes. The *VEGF* –634 C/G polymorphism is most probably a functional polymorphism, since significantly higher VEGF serum levels have also been reported in healthy subjects with the CC genotype of the C(–634)G polymorphism compared to those with the other genotypes [8]. It was reported that a −634G>C substitution enhanced *VEGF* expression at both transcriptional and translational levels [18]. Additionally, fasting serum VEGF levels in 104 diabetics with PDR were significantly higher from those of 29 controls without diabetes. Several environmental and genetical factors (hypoxia, hyperglycemia, oxydative stress, ischemia, *VEGF* gene polymorphism) influence serum VEGF levels [8,19].

In our study, vitreous VEGF levels were found to be affected by genetic factors (*VEGF* –634 C/G polymorphism) and by local factors in the eyes (extent of laser photocoagulation). Vitreous VEGF levels were affected by the *VEGF* –634 C/G polymorphism. Vitreous VEGF levels were significantly higher in 12 PDR patients with the CC genotype compared to 56 PDR patients with the other (CG + GG) genotypes. In our study as well as in other studies it was demonstrated that in PDR the extent of laser photocoagulation significantly affects VEGF level [1,4].

Vitreous VEGF levels were statistically higher in PDR eyes in comparison to the control group without diabetes, which is in agreement with other studies [1,3-5]. In our study, however, the levels of VEGF were higher than in other studies. The reason for this discrepancy is probably due to different methods of VEGF detection [20]. Moreover, vitreous VEGF levels were significantly higher in 12 PDR patients with the CC genotype compared to 56 PDR patients with the other (CG + GG) genotypes. We speculate that vitreous VEGF levels were also found to be affected by genetic factors.

Significantly higher vitreous VEGF levels were demonstrated in the eyes with macular edema compared to the eyes without macular edema. Our findings are in accordance with the findings of Funatsu and coworkers [4]. They demonstrated elevated vitreous VEGF in nonproliferative diabetic macular edema [1,3-5]. Moreover, vitreous levels of VEGF were elevated in active neovascularization in PDR that is in agreement with other reports [1,21].

VEGF is an important mediator in pathogenesis of diabetic retinopathy [8,13,18,22]. In the present study we demonstrated that the *VEGF* –634 C/G polymorphism affects the vitreous and serum levels of VEGF in patients with PDR, whereas the association, on the other hand, has not been demonstrated between the *VEGF* –634 C/G polymorphism and PDR. We speculate that other factors are more important than the *VEGF* –634 C/G polymorphism in the pathogenesis of advanced form of PDR (our group of patients with PDR in whom vitrectomy was performed).

In conclusion, VEGF is an important cytokine in PDR. The serum and vitreous VEGF levels were statistically higher in PDR eyes in comparison to the control group. Despite the effect of the *VEGF* –634 C/G polymorphism on serum and vitreous levels of VEGF in PDR, we found the *VEGF* –634 C/G polymorphism failed to contribute to the genetic susceptibility to PDR.
